# The Geography of the Alzheimer’s Disease Mortality in Spain: Should We Focus on Industrial Pollutants Prevention?

**DOI:** 10.3390/healthcare5040089

**Published:** 2017-11-25

**Authors:** Èrica Martínez-Solanas, Montse Vergara-Duarte, Miquel Ortega Cerdà, Juan Carlos Martín-Sánchez, Maria Buxó, Eduard Rodríguez-Farré, Joan Benach, Glòria Pérez

**Affiliations:** 1 ISGlobal, Center for Research in Environmental Epidemiology, 08003 Barcelona, Spain; 2 Health Inequalities Research Group, Employment Conditions Knowledge Network (GREDS-EMCONET), Department of Political and Social Sciences, Universitat Pompeu Fabra, 08005 Barcelona, Spain; montse.vergara@upf.edu (M.V.-D.); joan.benach@upf.edu (J.B.); 3 ENT Foundation, 08800 Vilanova i la Geltrú, Spain; mortega@ent.cat; 4 Biostatistics Unit, Department of Basic Sciences, Universitat Internacional de Catalunya, Sant Cugat del Vallès, 08195 Barcelona, Spain; juancarlosmarsan@gmail.com; 5 Institut d’Investigació Biomèdica de Girona, IDIBGI, Parc Hospitalari Martí i Julià, 17190 Salt, Spain; mbuxo@idibgi.org; 6 Environmental Toxicology, Barcelona Institute of Biomedical Research, CSIC-IDIBAPS and CIBER of Epidemiology and Public Health (CIBERESP), 08036 Barcelona, Spain; eduardo.rodriguez.farre@csic.es; 7 The Johns Hopkins University-Universitat Pompeu Fabra Public Policy Center, 08005 Barcelona, Spain; 8 Grupo de Investigación Transdisciplinar sobre Transiciones Socioecológicas (GinTRANS2), Universidad Autónoma de Madrid, 28049 Madrid, Spain; 9 Agència de Salut Pública de Barcelona—Public Health Agency of Barcelona, 08023 Barcelona, Spain; gperez@aspb.cat

**Keywords:** Alzheimer’s disease, mortality, industry

## Abstract

Alzheimer’s disease (AD) has a high worldwide prevalence but little is known about its aetiology and risk factors. Recent research suggests environmental factors might increase AD risk. We aim to describe the association between AD mortality and the presence of highly polluting industry in small areas in Spain between 1999 and 2010. We calculated AD age-adjusted Standardized Mortality Ratio (SMR), stratified by sex, grouped by industrial pollution density, compared for each small area of Spain. In the small areas with the highest mortality, the SMR among women was at least 25% greater than the national average (18% in men). The distribution of AD mortality was generally similar to that of high industrial pollution (higher mortality in the north, the Mediterranean coast and in some southern areas). The risk of AD mortality among women was 140% higher (123% among men) in areas with the highest industrial density in comparison to areas without polluting industries. This study has identified a geographical pattern of small areas with higher AD mortality risk and an ecological positive association with the density of highly polluting industry. Further research is needed on the potential impact of this type of industry pollution on AD aetiology and mortality.

## 1. Introduction

Alzheimer’s disease (AD) is the most common type of dementia [1]. Little is known about the aetiology of AD and its associated risk factors. Recent research suggests multiple potentially modifiable risk factors, e.g., a well-balanced diet and moderate alcohol consumption could delay cognitive decline and reduce AD risk [2,3], while obesity, overweight and environmental exposure might increase AD risk [2,4]. Some studies have suggested that exposure to chemical contaminants such as aluminium, mercury, lead and pesticides increase the risk of neurodegenerative disease [5,6]. This study is aimed to describe the relationship between AD mortality and the presence of polluting industries in small areas of Spain for the period 1999–2010.

We conducted an ecological study of AD-mortality and industrial polluting exposure in 2218 small areas of Spain (municipalities or aggregated municipalities) from the period 1999–2010. The methodology to build small areas has been further described in previous studies [7]. 

We obtained, by sex and 5-year age group, AD death counts (code G30 of the 10th revision of the International Classification Diseases) aggregated for a 3-year time-period and the 2001 census population counts. Levels of industrial exposure were estimated by the number and the presence (yes/no) of facilities register in the Spanish Pollutant Release and Transfer Register (PRTR) [8] for 2010 in each of the 2218 small areas. An indicator of the “density of polluting industries” (DPI) for each small area was created as the ratio between the number of facilities and the small-area surface per 100 km^2^. The DPI was categorized into four groups: areas without facilities and three groups corresponding to tertiles of the DPI distribution: 0.01–1.03; 1.04–4.04; 4.05–195 facilities/100 km^2^.

Age-adjusted AD-mortality risk by sex were estimated using Standardized Mortality Ratio (SMR) with an empirical Bayes approach [9]. We assumed a Poisson distribution model, using a random effect for small areas model, and including DPI as an explanatory variable. We obtained the SMR and its 95% confidence interval (CI) for AD mortality for each level of industrial density, in comparison with the reference category “without industrial facilities”.

## 2. The Geographical Distribution of Alzheimer’s Disease Mortality and Industrial Pollution

**Figure 1:**
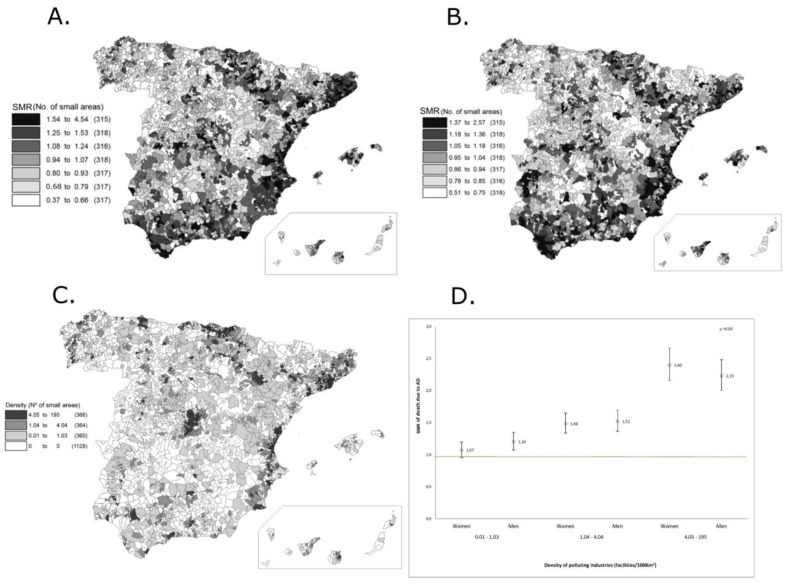
Maps of Alzheimer’s disease (AD) standardized mortality ratios (SMR) for women (**A**) and men (**B**) in Spain, 1999–2010. Density of polluting industries in small areas in Spain, 2010 (**C**). Age-and industrial density-adjusted Standardized Mortality Ratio (SMR) (95% confidence interval (CI)) of death due to AD for each sex in Spain, 1999–2010 (**D**). Reference level/category: without facilities.

Between 1999 and 2010 we observed 4,479,142 deaths in Spain (47.9% women and 52.1% men). The distribution by sex in Spain showed a larger bias towards men in mortality, compared to the overall European pattern, in which, for the same period, women registered only slightly less percentage of deaths than men (49.9% women and 50.2% men). Of the total of deaths registered in Spain, 100,637 deaths were from AD (69% women). Figure 1A and Figure 1B show the AD SMR for women and men in Spain. In the areas with the highest mortality, the SMR among women was at least 25% greater than the national average (18% in men). We detected a clear aggregation of areas with higher mortality in the northern regions, the Mediterranean coast, and in the transverse axis from the north to the east region, along the Ebro River. We also observed some areas with higher AD mortality in the south (especially in the west), and in the centre of Spain. In contrast, lower AD mortality was observed in the north and northwest and in some central areas. The geographical distribution pattern of the SMR was similar in both sexes. Among men, we also observed a higher mortality in the southeast, the Canary Islands and in some central areas.

In 2010, the Spanish Pollutant Release and Transfer Register (PRTR) contained information on about 6134 polluting industries located in 1403 small areas in Spain (49.1% of small areas had at least one facility). The distribution of the density of polluting industries presented considerable variability throughout the country (ranging from 0 to 195 facilities per 100 km^2^). The highest industrial density was located in the north-eastern area of the Lower Ebro Valley and along the Mediterranean coast (Figure 1C). Other large geographical areas with an aggregation of high industrial density were in the centre and the southwest of the peninsula, as well as the Canary Islands. 

## 3. The Association between Alzheimer’s Disease Mortality and Industrial Pollution Exposure

Figure 1D shows the aged-adjusted SMR (and 95% CI) of death due to AD for each DPI category in comparison with areas without industry. We observed a statistically significant association between AD-mortality and the density of polluting industry among both women and men (*p*-value < 0.05). The SMR increased in parallel with the density of highly polluting industry, most remarkably among women. In comparison to areas without polluting industries, the risk of death due to AD was 7% higher (20% in men) in areas with industrial density between 0.01 and 1.03 facilities/100 km^2^, 48% higher (52% in men) in areas with 1.04–4.04 facilities/100 km^2^, and more than 140% higher (123% in men) in areas with the highest industrial density. No statistically significant difference was observed among women for the lowest density category.

## 4. Implications for Further Research

Regions with the highest AD-mortality coincide with those historically industrialized, located in the north (mainly mining, iron and steel industry), centre (mostly chemical and metallurgical industries), the northeast of Spain (mainly textile, chemical and paper industries) as well as along the Mediterranean coast and in the south of the country.

Some studies suggest that high and prolonged exposure to chemical compounds and metals may accelerate the formation of senile plaques in the brain, increase oxidative stress, alter calcium homeostasis and promote neuronal death, all of which are common characteristics among AD patients [5]. For example, some toxic metals and other compounds generated by industry and agriculture, such as pesticides, mercury, cadmium or radioactive materials, are present at different levels along the course of the Ebro River valley [10]. In addition, some studies in Spain have evaluated the health impacts of industrial activity on diseases such as leukemia, bladder and lung cancers [11] and Parkinson’s disease [12]. More recently, a study highlighted that cancer mortality in Spain is around 17% higher in industrialized municipalities [13]. Most have mainly focused on industrial activity in the north and northeast, and more recently in the southwest of the country. In particular, our work highlights the fact that industries related to water supply, wastewater activity and waste management, as well as manufacturing industries were widely present in most areas in the top deciles of AD mortality (data not shown). Hence, new research should focus on these areas in order to thoroughly investigate the types of pollutants emitted by those industries.

We have considered AD-mortality stratified by sex for different reasons. Firstly, AD-mortality rate is higher among women [14]. Also, because we expected some differences in the geographical patterns for high and low-risk areas when analyzing by sex. Furthermore, it allows us to analyze separately the possible common risk factor of industrial pollution. A different AD-mortality distribution by sex could establish future hypotheses about other potential risk factors. For instance, the relationship between AD-mortality risk and occupational exposure through toxic compounds in the workplace among men [15].

Finally, some environmental questions should be taken into account for future research. First, some studies suggest that environmental pollution, as a broader concept including pollution from traffic or other sources, might be a potential risk factor for AD. Unfortunately, there are no similar sources of information for industrial facilities with lower levels of pollution than the thresholds established in the PRTR-based Spanish legislation, and existing sources of information for diffuse polluting sources are of much lower quality. Second, it is difficult to measure environmental risk factors individually, although ecological studies are one of the best epidemiological designs for analyzing how some risk factors operate at population level. Our results are limited due to its ecological design, which could be bias due to ecological fallacy. However, these kind of studies are useful to explore some hypotheses which are difficult to assess with other designs. We should also consider the fact that industrial exposure is determined by numerous other factors, such as socioeconomic status or cultural determinants. In this sense, some research reveals that under-privileged people and disadvantaged communities tend to live nearer to environmentally hazardous facilities [16], leading to environmental inequalities.

## 5. Public Health Policies Recommendations

To the best of our knowledge, this study is the first reporting the ecological association between AD-mortality and exposure to industrial pollution in small geographical areas. We have identified a geographical pattern of small areas with higher risk of death due to AD than the national average. Areas with a higher density of highly polluting industry had higher mortality due to AD compared with areas without polluting industries. Theses inequalities were greatest among women. These results could allow more efficient allocation of health care resources, as well as appropriate implementation of specific interventions adapted to the needs of each zone. Policies to control and decrease current levels of industrial pollution might play a role in AD mortality.

## References

[1] Wimo A., Prince M. (2010). World Alzheimer Report 2012 The Global Economic Impact of Dementia.

[2] Alzheimer’s Association (2012). 2012 Alzheimer’s disease facts and figures. Alzheimers Dement. J. Alzheimers Assoc..

[3] Campdelacreu J. (2012). Parkinson disease and Alzheimer disease: Environmental risk factors. Neurología (Barc. Spain).

[4] Di Carlo A., Baldereschi M., Amaducci L., Lepore V., Bracco L., Maggi S., Bonaiuto S., Perissinotto E., Scarlato G., Farchi G. (2002). Incidence of dementia, Alzheimer’s disease, and vascular dementia in Italy. The ILSA Study. J. Am. Geriatr. Soc..

[5] Moulton P.V., Yang W. (2012). Air pollution, oxidative stress, and Alzheimer’s disease. J. Environ. Public Health..

[6] Mutter J., Curth A., Naumann J., Deth R., Walach H. (2010). Does inorganic mercury play a role in Alzheimer’s disease? A systematic review and an integrated molecular mechanism. J. Alzheimers Dis..

[7] Benach J. (2004). Atlas de Mortalidad en Áreas Pequeñas en Cataluña (1984–1998).

[8] (2012). PRTR España. http://www.prtr-es.es/.

[9] Elliott P., Cuzick J., English D., Stern R. (1996). Geographical and Environmental Epidemiology: Methods for Small-Area Studies.

[10] Veses O., Mosteo R., Ormad M.P., Ovelleiro J.L. (2012). Potential toxicity of polycyclic aromatic hydrocarbons and organochlorine pesticides in sediments from the Ebro River basin in Spain. Bull. Environ. Contam. Toxicol..

[11] Buxó Pujolràs M. (2012). Descripció, Evolució i Possibles Causes de la Mortalitat al Sud-oest d’Espanya: Una Anàlisi des de L’epidemiologia Geográfica.

[12] Caudle W.M., Guillot T.S., Lazo C.R., Miller G.W. (2012). Industrial toxicants and Parkinson’s disease. Neurotoxicology.

[13] Fernández-Navarro P., García-Pérez J., Ramis R., Boldo E., López-Abente G. (2017). Industrial pollution and cancer in Spain: An important public health issue. Environ. Res..

[14] Wortmann M. (2012). Dementia: A global health priority-highlights from an ADI and World Health Organization report. Alzheimers Res. Ther..

[15] Santibáñez M., Bolumar F., García A.M. (2007). Occupational risk factors in Alzheimer’s disease: A review assessing the quality of published epidemiological studies. Occup. Environ. Med..

[16] Viel J.F., Hägi M., Upegui E., Laurian L. (2011). Environmental justice in a French industrial region: Are polluting industrial facilities equally distributed?. Health Place.

